# Emerging graphene derivatives as active 2D coordination platforms for single-atom catalysts

**DOI:** 10.1039/d2nr03453k

**Published:** 2022-09-07

**Authors:** Vítězslav Hrubý, Dagmar Zaoralová, Miroslav Medveď, Aristeidis Bakandritsos, Radek Zbořil, Michal Otyepka

**Affiliations:** Regional Centre of Advanced Technologies and Materials, Czech Advanced Technology and Research Institute (CATRIN), Palacký University Olomouc Šlechtitelů 27 783 71 Olomouc Czech Republic michal.otyepka@upol.cz; Department of Physical Chemistry, Palacký University Olomouc 17. listopadu 12 771 46 Olomouc Czech Republic; IT4Innovations, VŠB–Technical University of Ostrava 17. listopadu 2172/15 708 00 Ostrava-Poruba Czech Republic; Centre of Energy and Environmental Technologies, Nanotechnology Centre, VŠB–Technical University of Ostrava 17. listopadu 2172/15 708 00 Ostrava-Poruba Czech Republic

## Abstract

Single-atom catalysts (SACs) based on graphene derivatives are an emerging and growing class of materials functioning as two-dimensional (2D) metal-coordination scaffolds with intriguing properties. Recently, owing to the rich chemistry of fluorographene, new avenues have opened toward graphene derivatives with selective, spacer-free, and dense functionalization, acting as in-plane or out-of-plane metal coordination ligands. The particular structural features give rise to intriguing phenomena occurring between the coordinated metals and the graphene backbone. These include redox processes, charge transfer, emergence, and stabilization of rare or otherwise unstable metal valence states, as well as metal–support and metal–metal synergism. The vast potential of such systems has been demonstrated as enzyme mimics for cooperative mixed-valence SACs, ethanol fuel cells, and CO_2_ fixation; however, it is anticipated that their impact will further expand toward diverse fields, *e.g.*, advanced organic transformations, electrochemical energy storage, and energy harvesting.

## Abstract 2

Single-atom catalysts (SACs) based on graphene derivatives are an emerging and growing class of materials functioning as two-dimensional (2D) metal-coordination scaffolds with intriguing properties.

## Graphical abstract

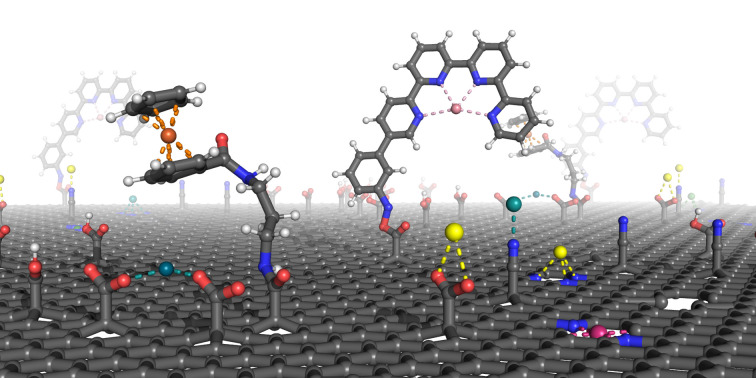

## Introduction

Today, 80% of all commercially available chemical products require catalysts in some stage of their manufacture.^1^ Most of the processes involve heterogeneous catalysts due to their recyclability. The main drawback of heterogeneous catalysts is that only a small fraction of active centers is accessible for a catalyzed reaction.^2^ In order to tackle this disadvantage, single-atom catalysts (SACs) are being developed which have active sites consisting of single metal atoms immobilized on a support.^3,4^ Therefore, SACs exhibit ultimate atom utilization efficiency while being easily recyclable.^2,3^ Utilizing SACs for the production of technologically and commercially valuable products will ultimately reduce their cost and, most importantly, will help save natural resources of precious metals.^5,6^

The development of SACs requires supports that effectively anchor single metal species (SMSs) responsible for the catalytic activity. Such effective anchoring should lead to SMSs in targeted redox and spin states, strongly interacting with the support to prevent unwanted migration and agglomeration of SMSs. Metal alloys, oxides, carbides, nitrides, sulfides, and carbon nanomaterials have been identified as suitable supports.^1,7–10^ Carbon-based nanomaterials are particularly attractive because they are lightweight and composed of abundant and non-toxic elements, and offer high surface area and conductivity.^11^ Recently, 2D carbon-based materials, *i.e.*, graphene and its derivatives, have been identified as promising supports due to their large surface area, high affinity towards SMSs, high electric conductivity attractive for the electrocatalytic processes, and good stability. Furthermore, graphene enables efficient charge transport between the metal atom and the support, thus enhancing the catalytic activity^10^ or even conditioning the catalytic behavior of the atom for particular reactions.^12^ To effectively anchor SMSs, the presence of heteroatoms such as N, O, and S in the graphene support is crucial.^10^ So far, mainly graphene defects containing heteroatoms, particularly N-doped graphene, have been studied and utilized as SACs supports.^13–15^ Nevertheless, it is very challenging to gain control over the nature of the graphene defects. Recently, it has been proposed that materials containing graphene grafted with functionalities enabling selective coordination of metal atoms through chemical functionalization could be superior SAC substrates.^1^

### Graphene functionalization

Due to its high chemical inertness, direct functionalization of graphene remains a challenging task. Except direct hydrogenation and fluorination of graphene, which leads to the formation of the only two stoichiometric graphene derivatives, *i.e.*, graphane^16^ and fluorographene,^17,18^ a limited set of reactions involving pristine graphene such as cycloaddition^19–21^ and nucleophilic addition^22^ is known to offer graphene derivatives with more complex and defined functionalities. By contrast, graphene oxide (GO) prepared by harsh oxidation of graphite has received considerable attention over the past decade due to its cheap and affordable synthesis with mass production potential and the presence of a high number of oxygen-bearing groups, whose reactivity makes its further functionalization feasible.^23^ The nature of GO is, however, rather complex owing to the presence of various oxygen functions, with the resulting character of GO depending on the particular procedure of its synthesis.^24^ Moreover, a recent review on the utilization of GO as a support for the development of SACs has not covered controllable functionalization of GO with out-of-plane functionalities, focusing instead on its transformation into specific forms of N-doped graphene with anchored SMSs.^23^ In addition, GO is not an electrically conductive nanomaterial,^25^ which limits its application in electrocatalysis. GO can be (thermally or chemically) reduced to regain electrical conductivity,^26^ but such modifications are accompanied by the loss of significant amounts of functionalities.

Thanks to its reactivity,^17,27^ fluorographene (FG) was proposed as a handy starting material for controllable^28^ and scalable synthesis of highly functionalized graphene derivatives.^29^ The most straightforward options for FG synthesis are direct fluorination of graphene by gaseous elemental fluorine^30^ or XeF_2_^18,31,32^ at elevated temperature, or by plasma treatment in the presence of elemental fluorine,^33^ CF_4_,^34^ fluoroform,^35^ or SF_6_.^36^ Additionally, photoinduced decomposition of fluoropolymer on graphene allows its patterned fluorination.^37^ Obtained FG can be applied in electronics due to its electro-insulating properties,^38^ as an electrode separator,^39^ as a matrix in MALDI mass spectrometry,^40^ in composites with perovskites for solar cells,^41^ and as coatings for its hydrophobicity.^42^ Nevertheless, FG used for the synthesis of graphene derivatives is mostly prepared by chemical exfoliation of graphite fluoride in a variety of organic solvents under sonication, which is a cheap, simple, and scalable method.^29^ Created well-processable FG dispersions can be directly reacted with various nucleophilic reagents under mild conditions in order to introduce a desirable group/molecule/functionality on a graphene platform with high control over the material stoichiometry and topology.^29^ Graphene derivatives prepared this way have numerous advantages over the materials derived from the extensively used graphene oxide. Their electric conductivity^43^ makes them applicable in electrocatalysis.^44^ They display good processability in the form of dispersions in a variety of solvents. They also offer a broad portfolio of homogeneously distributed functionalities covalently grafted onto the graphene lattice, and their further functionalization *via* the chemistry of the attached functionalities.^45^ Thanks to these properties, FG-derived materials can be efficiently utilized as supporting materials for SACs^12,44–48^ because introduced functional groups can immobilize SMSs and avoid undesirable processes such as the formation of larger metal clusters and the leaching of metal particles during reactions.

### Fluorographene reactivity

**Fig. 1:**
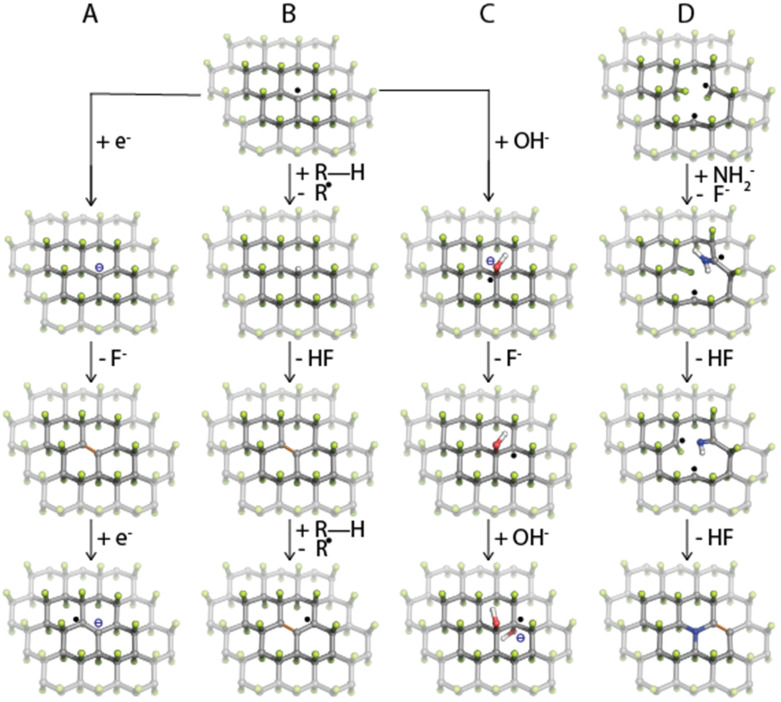
The first steps of possible reaction pathways of FG defluorination, nucleophilic substitution, and heteroatom doping that start on a radical center in FG. (A) Electron transfer, (B) hydrogen transfer, (C) nucleophilic substitution, and (D) heteroatom doping. Carbon atoms are grey, fluorine green, oxygen red, nitrogen blue, and hydrogen white. C=C double bonds are orange.

In one of the pioneering studies on the fluorination of graphene, Nair and coworkers^49^ observed strong paramagnetism in highly-fluorinated graphene samples, ascribing it to radical point defects (*i.e.*, carbon sites with one missing fluorine ad-atom) that naturally form during the fluorination of graphene. Later, by combining the electron paramagnetic resonance (EPR) technique with density functional theory (DFT) calculations, Medveď *et al.*^50^ demonstrated that such radical sites play a crucial role in FG chemistry. Owing to their high electron affinity, they can accept an electron into the SOMO orbital and initiate the defluorination process even in the presence of mild reducing agents (Fig. 1A), which was also confirmed experimentally by comparing several non-nucleophilic organic bases.^51^ Moreover, the FG radical centers can assist a homolytic R–H bond cleavage (Fig. 1B), as demonstrated by the reaction mechanism of hydrogen transfer from DMF to FG radical defect.^50^ The formed DMF· radical could then attack fluorine ad-atoms of FG to form *N*,*N*′-dimethylcarbamoyl fluoride and to recreate a radical on FG, thus promoting further FG defluorination.

**Fig. 2:**
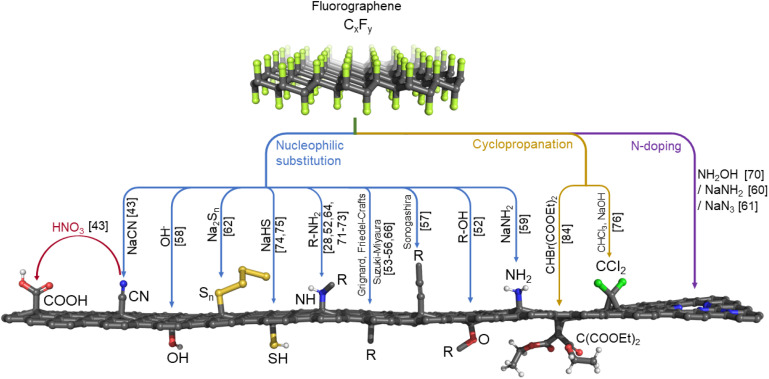
Overview of possible ways of FG's direct derivatization. The numbers in the brackets refer to literature as listed in the references.

Thanks to the electron-withdrawing ability of the surrounding fluorine ad-atoms, the radical sites of FG have a strong electrophilic character. They can be attacked by various nucleophiles such as NH^−^_2_, CN^−^, OH^−^, organic amines or alcohols,^28,52^ and Grignard reagents,^53,54^ or it may undergo Friedel–Crafts reaction,^55^ and Suzuki-Miyaura^56^ or Sonogashira couplings.^57^ This way, a wide variety of functionalities can be grafted onto the surface of graphene (Fig. 2). The reaction may concurrently proceed along the three reaction pathways displayed in Fig. 1A–C. Nevertheless, the different kinetics and thermodynamics of the individual steps offer the possibility of controlling the degree of defluorination and substitution by the choice of the solvent, the nucleophilic agent, the reaction time, and the conditions. Indeed, Tuček *et al.*^58^ synthesized a series of five hydroxofluorographenes of different compositions by changing the –OH-containing precursors, the reaction time, and the temperature. Similarly, Zhao *et al.*^59^ synthesized derivatives co-functionalized by various amounts of –F and –NH_2_ groups while changing the reaction conditions. Since radical centers also occur on the edges of vacancies, they play a vital role in the mechanism of FG nitrogen doping (Fig. 1D).^60,61^

It is worth noting that not only radical point defects but also larger defluorinated areas like defluorinated chains, rings, or islands can be attacked by nucleophiles, as demonstrated in the case of covalent grafting of polysulfide chains to the surface of partially fluorinated graphene ((p)FG).^62^ The negative charge that is brought to a partially or fully fluorinated graphene by the nucleophile initiates a fluoride anion release.^28,60^ In combination with frequently used solvents that promote (p)FG defluorination, such as DMF, NMP, acetone, and DMAc, covalent functionalization of (p)FG is often accompanied by either partial or complete defluorination of the material.^27,43,52,53,58,63,64^ On the other hand, in some cases, extensive defluorination of the material may be undesirable since it lowers the intrinsic self-lubricating ability of FG. This can be tackled by activating the dormant radicals in pFG, as demonstrated by acrylic acid and styrene, which bind to the pFG surface without losing fluorine ad-atoms.^65^ Due to their electron-withdrawing effect, residual F atoms make the prepared derivative act as a dienophile in Diels–Alder cycloaddition reactions, enabling to heterobifunctionalize graphene with functionalities that are otherwise impossible to graft at the same time.^66^ Nevertheless, tunable one-step double- or multi-functionalization of FG based on the nucleophilicity of the grafted molecules using nucleophiles is also feasible.^67^

Recently, a slightly different reaction mechanism of FG functionalization was proposed by Siedle *et al.*^68^ Successful attachment of nitrogen-containing functional groups to the surface of graphite fluoride and simultaneous defluorination of the material deploying benzylamine and tetramethylethylenediamine was explained as a so-called proton-coupled electron transfer. Interestingly, it was demonstrated that FG radical centers fostered the formation of iminium ion fragments from benzylamine and caused fragmentation of tetramethylethylenediamine. The resultant nitrogen moieties were able to attach to C=C bonds in pFG or recombine in order to form various amines. A similar conclusion was achieved for the reaction of FG with NaNH_2_ in DMF solvent.^60^ The GC-MS analysis of the supernatant in combination with DFT calculations showed that FG radical centers allowed a variety of radical side reactions, leading to a number of possible by-products of the reaction.

### Graphene derivatives achieved *via* the chemistry of fluorographene

A plethora of FG derivatives (see Fig. 2) with broad applicability has been synthesized through the principles of FG chemistry explained above.^69^ A material based on FG modified with hydroxyl (–OH) groups that enabled a superexchange interaction between the spins of the parent FG was identified as the first organic material with room-temperature antiferromagnetic ordering with a transition into ferromagnetic regime at low temperatures.^58^ Spin-rich N-doped graphene (NG) prepared from FG using hydroxylamine exhibited spin-switch behavior under microwave radiation.^70^ Regarding energy storage applications, a sulfur-chain-functionalized graphene acted as a high-capacity cathode material for lithium–sulfur batteries.^62^ An N-doped derivative employing sodium azide exhibited unprecedentedly high capacitance with power density up to 200 Wh L^−1^ at a power of 2.6 kW L^−1^ in symmetric supercapacitors.^61^ Generally, as FG has been recognized for its susceptibility to reactions with organic amines,^28,64^ the resulting products of reactions of FG with 5-aminoisophtalic acid or arginine amino acid were tested as supercapacitor electrode materials with promising results.^71,72^ Controllable functionalization of FG with n-octylamine molecules allowed tailoring its nonlinear optical properties.^73^ As for the bio-applications of FG derivatives, the ability of thiol-functionalized FGs through the reaction with sodium hydrosulfide^74^ or *via* reaction with xanthogenate ion and subsequent hydrolysis of the prepared material^75^ to adsorb biomolecules, *e.g.*, nucleic acids and enzymes, can be utilized in biosensing and environmental remediation. Modification of FG with chlorine atoms was achieved by immobilizing dichlorocarbene on the FG.^76^

Cyanographene (GCN),^43^ a nitrile groups-carrying material created by the substitution of FG's fluorine atoms with –CN groups using sodium cyanide, is among the most versatile FG-derived materials. Such functionality can anchor transition metal atoms, which can be utilized in SACs.^12,47^ It also creates a strong bond with silver nanoparticles, which hampers the defense mechanism of silver-resistant bacteria.^77^ The nitrile groups in GCN can be readily hydrolyzed into carboxyl (–COOH) groups, thus forming graphene acid (GA)^43,78^ that may be also utilized as a ligand for anchoring transition metals^45^ or as a recyclable substrate for water remediation or retrieval of precious metals from waste.^79^ It is worth noting that GA was successfully applied as an electrode material in supercapacitors^80^ and lithium-ion batteries.^81^ Using carbodiimides, GA can be conjugated with primary amines, allowing further functionalization, which makes GA a robust substrate for the immobilization of biomolecules used as nano(bio)catalysts^82^ and biosensing platforms.^83^ A network of carboxyl groups on graphene can be also achieved through the Bingel reaction of malonate ester with FG and its subsequent hydrolysis. Its conjugation with iron phthalocyanine resulted in a material with unprecedented capacitance due to the formed zwitterionic network of residual –COOH groups and Fe ions.^84^

### Graphene derivatives-based SACs

**Fig. 3:**
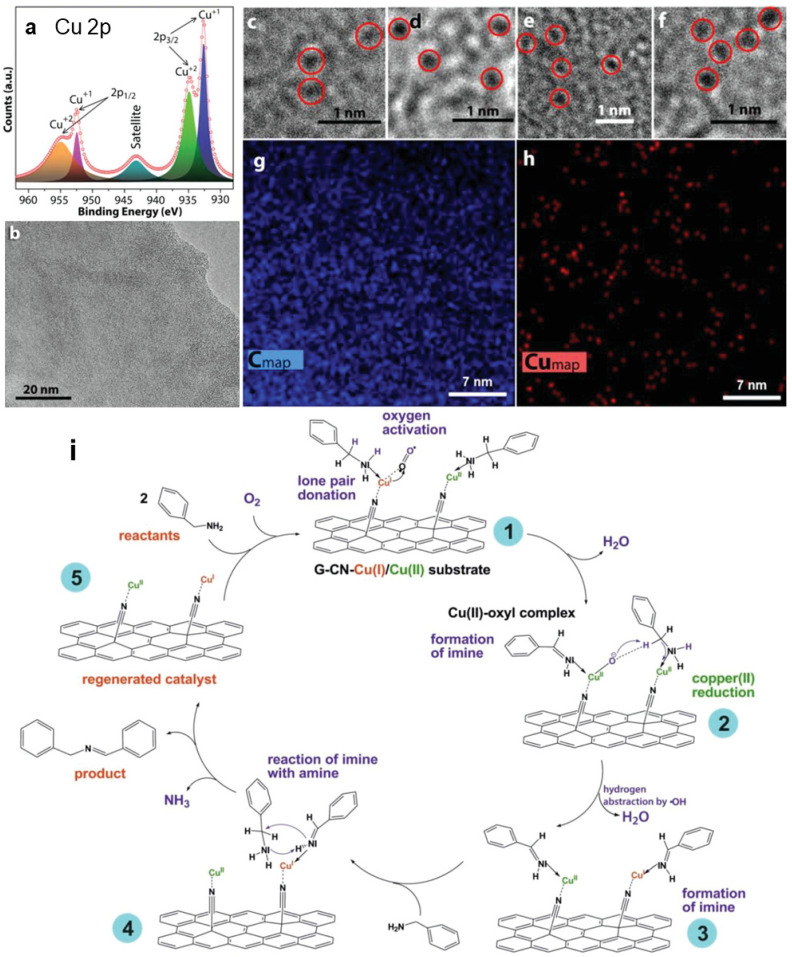
(a) HR-XPS Cu 2p spectrum of the Cu(ii)/Cu(i)⋯GCN catalyst. (b) HRTEM image of representative Cu(ii)/Cu(i)⋯GCN flake. (c–f) Representative high magnification HR-TEM images from the catalyst showing high-contrast spots originating from embedded copper atoms (c and d) before and (e and f) after the catalytic reaction. EDS chemical mapping of the Cu(ii)/Cu(i)⋯GCN catalyst for (g) C and (h) Cu. (i) Mechanism of the catalytic cycle of amine coupling reaction using Cu(ii)/Cu(i)⋯GCN catalyst. This figure has been adapted from ref. 12 with permission from the John Wiley and Sons, copyright 2019.

First-principles calculations in combination with experimental data have confirmed that among graphene derivatives bearing –F, –OH, –CN, and –H functional groups, GCN equipped with nitriles was the only one that was able to bind Pt atom without compromising the structure of the material.^46^ Bakandritsos *et al.*^12^ successfully immobilized Cu(ii) ions by nitrile groups of GCN (Fig. 3a and c–h). High-resolution X-ray photoelectron spectroscopy (HR-XPS), EPR, and X-ray absorption near edge structure (XANES) imaged the anchoring of Cu ions as SMSs, with a half of Cu(ii) ions being reduced to Cu(i) (Fig. 3a). Indeed, the DFT calculations indicated that the positive charge and spin density of Cu ion significantly decreased after anchoring to GCN. Owing to the present Cu(i) species, this mixed-valence Cu(ii)/Cu(i) catalyst was able to activate oxygen molecules and excelled in catalyzing an oxidative amine coupling (Fig. 3i) and selective benzylic C–H group oxidation of hydrocarbon derivatives. Possible generalization of mixed-valence SACs anchored to graphene derivatives was demonstrated on Fe(iii)/Fe(ii) cations immobilized on GA, detected by Mössbauer spectroscopy. The coordination of metal atoms or cations on the –CN and –COOH groups of GCN and GA is associated with a charge transfer between the substrate and SMS. The amount of the transferred charge correlates with the electron affinity of the bare metal cation and with the dissociation energy of the GCN/GA⋯Me bond.^47^ Interestingly, the analysis of spin populations of the metal atoms/cations anchored to GCN/GA demonstrated that their oxidation states were often the same despite their different initial charges. In the case of cation pairs Fe^2+^/Fe^3+^, Co^2+^/Co^3+^, and Cu^+^/Cu^2+^, this feature, in combination with high bond dissociation energy, could be used for designing new mixed-valence SACs. The ability of GCN to anchor metal cations was also discussed in the study of Kadam *et al.*^44^ on the successful design of Co-based SAC that has shown efficient and selective electrocatalytic activity toward hydrazine oxidation.

GA is another efficient supporting material for SACs and catalytically active metal nanoparticles.^45^ Blanco *et al.*^48^ succeeded in binding *in situ* formed ultrasmall (<1 nm) Pd nanoparticles to the –COOH groups of GA through a mild impregnation of GA with Pd(OAc)_2_ in acetonitrile. Such GA⋯Pd nanohybrids exhibited high catalytic activity in the Suzuki-Miyaura cross-coupling reaction under environmentally friendly conditions.^48^

**Fig. 4:**
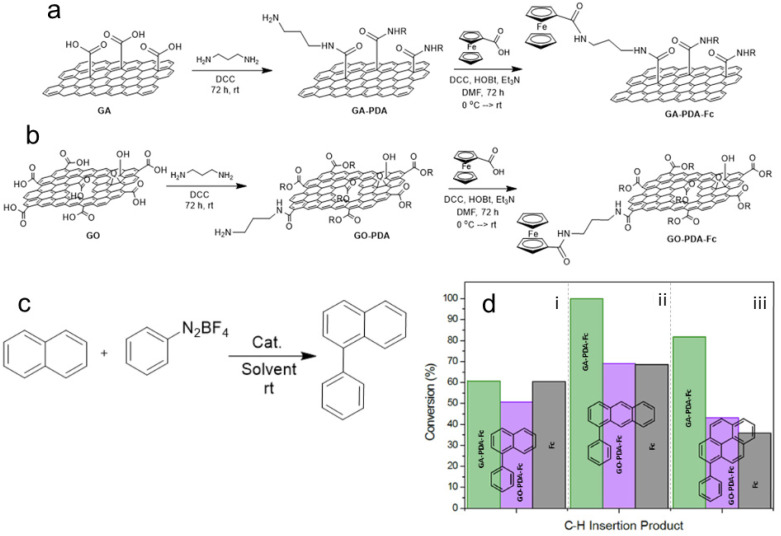
(a) Heterogenization of ferrocene (Fc) on GA modified with 1,3-diaminopropane (PDA) and (b) on PDA-modified GO. (c) Model reaction of arene C–H insertion of benzendiazonium tetrafluoroborate on naphthalene catalyzed using the tested ferrocene-based catalysts. (d) Comparison of catalytic activities of GA-PDA-Fc (green bar), GA-PDA-Fc (magenta), and molecular Fc (grey) on three different substrates; (i) naphthalene, (ii) anthracene, and (iii) pyrene. This figure has been adapted from ref. 85 with permission from the Elsevier, copyright 2018.

FG-derived graphene derivatives can be also used to anchor homogeneous catalysts, thus improving their stability and recyclability while their superior activity and selectivity remain preserved. Mosconi *et al.*^85^ reported a successful covalent functionalization of GA and GO with ferrocene (Fc) by conjugating both species with 1,3-diaminopropane (PDA) (Fig. 4a and b). The resultant materials were active and recyclable catalysts for the C–H insertion reaction of benzenediazonium tetrafluoroborate on naphthalene and higher polycyclic aromatic hydrocarbons. It also confirmed prior concerns about the character of GO, whose functionalization was much poorer compared to the GA-based catalyst, which contained higher Fe amounts and thus exhibited better catalytic activity. In the model reaction using naphthalene as a substrate (Fig. 4c), the catalytic activity of the GA-PDA-Fc was essentially the same as that of the molecular ferrocene. Remarkably, GO and GA-based catalysts were more effective in catalyzing the reaction on substrates with a higher number of condensed aromatic rings compared to the molecular ferrocene (Fig. 4d). The better performance of the graphene-based materials was ascribed to the adsorption of such reactants onto the graphene support, which facilitated their access to the ferrocene functionality. Such a quality of the graphene support may motivate the community to further exploit graphene-based catalysts. The same group succeeded in binding cobalt quaterpyridine to GA and obtained a material very active in converting CO_2_ into CO or formate, which resembled artificial photosynthesis.^86^ GA may also be used as a scaffold for electrostatic immobilization of molecular catalysts, which was found to be applicable in the case of Ni-based complex, a catalyst for the reversible electrocatalytic hydrogen oxidation reaction.^87^ The discussion on the almost unexplored potential of GA for the utilization as a biocatalyst has been initialized in the study by Seelajaroen *et al.*^82^ dealing with the grafting of dehydrogenase enzyme to the carboxylic groups of GA.

Not only covalent functionalization but also heteroatom doping of graphene offers promising active sites for immobilizing SMSs.^88–92^ For instance, Fe and Co atoms anchored to the N-doped carbon framework are active catalysts for the oxygen reduction reaction (ORR), oxygen evolution reaction (OER), hydrogen evolution reaction (HER), and water splitting.^4,6,15,89,90^ However, to our best knowledge, there has been no report so far on Me⋯NG complexes synthesized using FG chemistry, even though FG is a promising precursor for the synthesis of NG with a tunable amount of nitrogen.^60,61,70,93,94^

## Summary and perspective

**Fig. 5:**
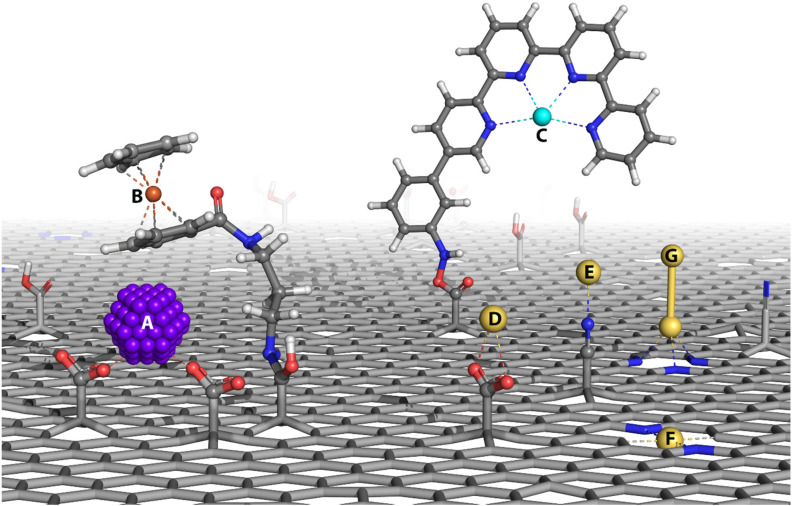
A depiction of so far investigated routes to anchoring SMSs for utilization as SACs to graphene derivatives. (A) Immobilization of nanoparticles by covalently grafted functional groups. Conjugation of carboxyl groups of GA with (B) ferrocene and (C) Co quaterpyridine metal complexes. Coordination of SMS to (D) carboxyl group of GA, (E) nitrile group of GCN. Anchoring of (F) SMS and (G) metal atom dimer to defects in the structure of graphene and nitrogen-doped graphene.

In summary, the chemistry of FG enables the design of well-defined 2D materials with covalently grafted functionalities capable of conjugating other molecules, including, *e.g.*, enzymes, anchor catalytically active SMSs, and metal complexes (Fig. 5). Such versatility, together with many valuable properties, including carbon-based structure, large surface area, and conductivity, make them particularly attractive for (electro)catalytic applications. Moreover, the high scalability of graphene-based support materials synthesis directly from graphite fluoride and the simplicity of SMS immobilization could be utilized for industrial production of graphene-based SACs. The usage of graphite fluoride also brings some challenges. The range of lateral dimensions of flakes of the prepared material may vary from nano- to micrometer scale depending on the precursor, synthesis, and purification methods. The prepared materials carry on homogeneously distributed functionalities, however the control over the resultant structure of the material cannot be achieved at atomic precision. Side reactions, *e.g.*, grafting of byproducts or solvent-borne contaminants to the FG, can affect the material's final composition.^43,60^ Nevertheless, the potential of SACs based on graphene derivatives was already demonstrated for oxidative amine coupling, selective benzylic C–H group oxidation, C–H insertion reactions, and an efficient and selective electrocatalysis of hydrazine oxidation. The catalysts displayed, besides their activity, also competitive stability, and recyclability. All these features predispose FG-based graphene derivatives to act as suitable platforms for SACs.

The potential of such materials can be further increased by advancing their properties and introducing new features. Controllable chemistry of FG allows balancing a functionalization degree and the material conductivity because the conductivity decreases with an increasing functionalization degree. Concerning the new features, stereospecific (chiral) catalysis has not yet been implemented in any of the reported SACs so far due to the challenging control over the close environment of the metal atom. The chemistry of FG has the potential to overcome such limitations of SACs by grafting ligand functionalities onto graphene, providing a well-defined coordination sphere for the immobilized metal atom. Moreover, the introduction of chiral functionalities to graphene could also imprint stereospecificity to make recyclable chiral catalysts. We therefore propose that the immobilization of proven homogeneous metal catalysts onto graphene, deploying the principles of the chemistry of FG and GA summarized in this work, can be a route to straightforward development of new recyclable and possibly regioselective and stereospecific graphene-based SACs. The new generation of SACs derived from the FG chemistry would also benefit from the control of the single-atom valence state, sophisticated control of the SA coordination, and the active role of graphene support in the reaction mechanism.

## Conflicts of interest

There are no conflicts to declare.

## Supplementary Material
